# Global minimization via classical tunneling assisted by collective force field formation

**DOI:** 10.1126/sciadv.abh1542

**Published:** 2021-12-22

**Authors:** Francesco Caravelli, Forrest C. Sheldon, Fabio L. Traversa

**Affiliations:** 1 Theoretical Division (T4), Los Alamos National Laboratory, Los Alamos, NM 87545, USA.; 2 Center for Nonlinear Studies, Los Alamos National Laboratory, Los Alamos, NM 87545, USA.; 3 London Institute for Mathematical Sciences, 35a South St., London W1K 2XF, UK.; 4 MemComputing Inc., San Diego, CA 92121, USA.

## Abstract

Simple elements interacting in networks can give rise to intricate emergent behaviors. Examples such as synchronization and phase transitions often apply in many contexts, as many different systems may reduce to the same effective model. Here, we demonstrate such a behavior in a model inspired by memristors. When weakly driven, the system is described by movement in an effective potential, but when strongly driven, instabilities cause escapes from local minima, which can be interpreted as an unstable tunneling mechanism. We dub this collective and nonperturbative effect a “Lyapunov force,” which steers the system toward the global minimum of the potential function, even if the full system has a constellation of equilibrium points growing exponentially with the system size. This mechanism is appealing for its physical relevance in nanoscale physics and for its possible applications in optimization, Monte Carlo schemes, and machine learning.

## Teaser

An alternative classical mechanism to quantum and thermal tunneling.

## INTRODUCTION

Tunneling and escape phenomena occur when a barrier separates two minima and a particle escapes (tunnels) from one minimum to the other aided by either spreading of the quantum wave function or via thermal fluctuations (*1*, *2*). The discovery of quantum tunneling was a paradigmatic shift from classical mechanics and underlies many important physical phenomena and technologies (e.g., alpha decay, scanning tunneling microscopy). Similarly, in thermal or stochastic dynamical systems, particles can escape from a metastable state when thermal fluctuations/noise impart sufficient energy to surmount the barrier (*2*). An analogy between these behaviors is expected considering that the Schrödinger equation can be mapped to a non-Markovian stochastic system (*3*) or to a classical particle in a nonlocal force field (*4*).

Escape phenomena are of current interest in computation, where tunneling is a leitmotif for optimization schemes both in classical and quantum frameworks such as simulated and quantum annealing (*5*, *6*). At the same time, there is mounting interest in alternative approaches to computation and optimization to meet rapidly growing demands on computing (*7*–*12*). Proposals using oscillators or frequency domain encoding (*8*, *11*–*14*), leveraging near- or in-memory computation (*9*, *15*–*18*), and memcomputing (*9*, *18*) are being used to more efficiently solve difficult problems across optimization (*19*, *20*).

Among these alternatives, interest in active or chaotic systems has experienced a revival (*21*). Chaotic dynamics have been posed as an obstacle to reaching fixed points in dynamical system-based computing (*22*), but there has also been evidence that unstable dynamics can improve the efficiency of some optimization schemes. In particular, unstable dynamics can lead to escaping local minima (*23*) in memristor-implemented simulated annealing. While escape phenomena are familiar in quantum and thermal settings, we are aware of no examples of classical dynamical systems, which exhibit barrier escapes when the system is a-thermal and passive.

Here, we present an example of barrier tunneling as an emergent, multiparticle effect in an a-thermal, passive (*24*) system moving in an effective potential. The effects of stochasticity in thermal systems or the spreading wave function in quantum systems are replaced by hidden degrees of freedom, which transition from a laminar phase (i.e., always negative local Lyapunov exponents) to an unstable or transiently unstable regime (i.e., local Lyapunov exponents positive for short transients).

## RESULTS

### The framework

**Fig. 1.**
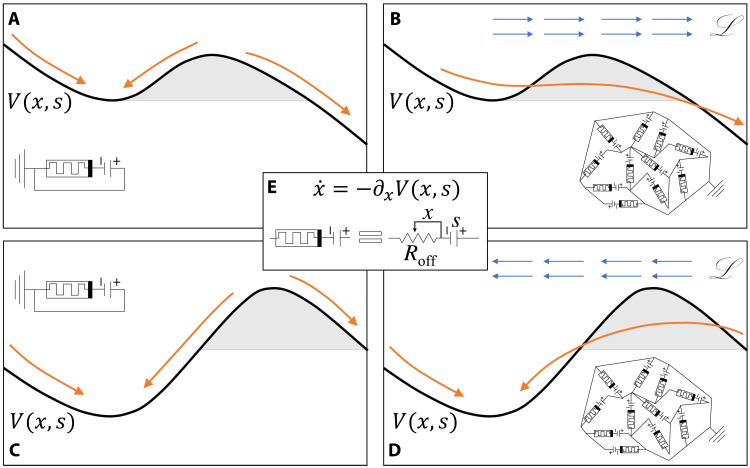
Representation of the tunneling phenomenon. On the left (**A** and **C**), the memristor potential *V*(*x*, *s*) for a single device is sketched when the applied voltage *s* takes high (A) and low (C) value. The directions that the internal memristor state *x* take, depending on the initial conditions, are shown in orange arrows, highlighting the two attractors. On the right (**B** and **D**), the same two settings are shown for a memristor network. The potential *V*(*x*, *s*) now governs the mean field state of the system. In this case, the emergent Lyapunov force (blue arrows) pushes the system toward the global minimum, creating an effective tunneling through the barrier. In the middle inset (**E**), the equivalent schematic of the memristive system is reported, highlighting the role of the potential *V*(*x*, *s*).

The system considered is inspired by the dynamics of memristor networks (*25*–*30*) that can be interpreted as flows on a graph, which are coupled through dynamics on the edge resistances. We study the equations of motion of the edges in which a low-dimensional effective potential representation shows barrier escapes. This property is shown in Fig. 1 and is the central result of this paper, which we characterize both analytically and numerically. The effect comes from the intrinsic coupling among elements, which can be thought of as induced by Kirchhoff’s laws. Leveraging the linearity of Kirchhoff’s laws to eliminate flow variables leads to a closed equation in which the edges interact nonlinearly through a matrix inverse, which, in turn, involves the state variables. Because of the nonperturbative nature of this coupling, we use a recently obtained exact large-*N* formula for the matrix inverse and analytically show that the effective coarse-grained dynamics can be approximated by that of a single memristor, i.e., a mean field approximation. As we show below, when the effective potential lacks convexity, the emergent Lyapunov force pushes the system into the absolute minimum of the potential, thus effectively tunneling through the barriers. This Lyapunov force is directly related to instabilities in the dynamics.

The dynamical equation for a circuit of memristors has been derived in (*31*). In particular, it models a flow network that obeys current and energy conservation and whose edge dynamics are bounded to an interval. A polar resistor with memory can be described by an effective dynamical resistance, which depends on an internal parameter *x*. For instance, TiO_2_ memristors are approximately described by the functional form *R*(*x*) = *R*_off_(1 − *x*) + *xR*_on_, where *R*_on_ < *R*_off_ are the limiting resistances, and the state variable *x* ∈ [0,1] physically describes the size of the oxygen-deficient conducting layer (*27*). The internal memory parameter evolves, to the lowest order of description, according to a simple equation of the form

$$\frac{d}{\mathrm{dt}}x=\frac{R_{\text{off}}}{\beta }I-\alpha x=\frac{R_{\text{off}}}{\beta }\frac{V}{R(x)}-\alpha x$$ (1)

with hard boundaries. The parameters α and β are the decay constant and the effective activation voltage per unit of time, respectively, and determine the time scales of the dynamical system.

The model above is the simplest description of a polar resistive device, and many extensions have been considered. For example, removing the hard boundaries and multiplying by a window function can approximate diffusive effects near the boundaries (*32*–*34*). Similarly, nonlinear conductive effects may be included by replacing *I* with a function *f*(*x*, *I*) or introducing new parameter dependencies, for instance, temperature in the case of thermistors (*35*). Comparisons between these models (*36*–*39*) show that many are more faithful to the precise *I-V* curves of physical devices, but most (if not all) share the basic pinched hysteresis phenomenology of the linear model. In analytical work, we assume that the dynamics are linear in the currents to demonstrate the behavior we study in the widest possible context. However, the behavior is quite robust, and in the Supplementary Materials, we show that it applies to many extensions of this equation including different window functions and conduction effects.

For a single memristor under an applied voltage *S*, we use Ohm’s law *S* = *RI* to obtain an equation for *x*(*t*) in adimensional units (τ = α*t*) given by

$$\frac{d}{d\tau }x=\frac{S}{\alpha \beta }\frac{1}{1-\chi x}-x=-\partial_{x}V(x,s)$$ (2)

Here, we have defined $\chi =\frac{R_{\text{off}}-R_{\text{on}}}{R_{\text{off}}}$ and $s=\frac{S}{\alpha \beta }$, with 0 ≤ χ ≤ 1 in the physically relevant cases and *V*(*x*, *s*) as an effective potential.

**Fig. 2.**
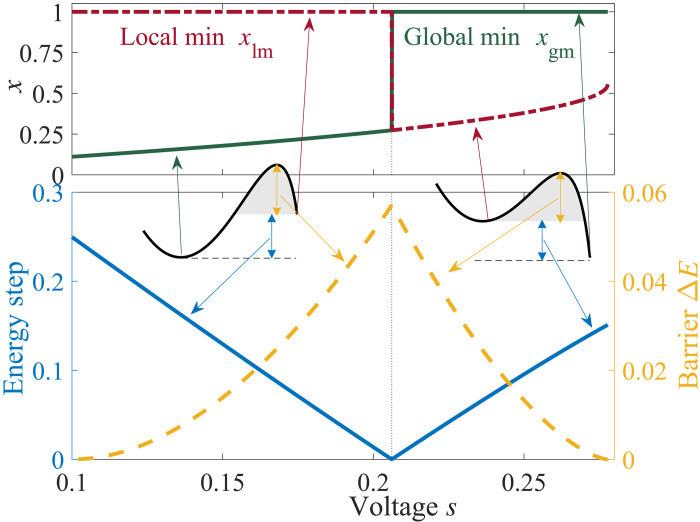
Structure of the local and global minima of the potential. **Top**: Local minimum (*x*_lm_; dashed-dotted red line) and global minimum (*x*_gm_; solid green line) locations are shown as function of voltage *s*. **Bottom**: Energy step (solid blue curve) and barrier Δ*E* (dashed yellow curve) as a function of voltage *s*.

The dynamics of the one-dimensional system, which is described by a single memristor device, is fully characterized by gradient descent in the potential

$$V(x,s)=\frac{1}{2}x^{2}+\frac{s}{\chi }\text{log}\;(1-\chi x)$$ (3)

with $s=\frac{S}{\alpha \beta }$. This potential can have two minima separated by a barrier as shown in Fig. 2 for various values of *s* and for χ = 0.9. We consider *s* on the restricted interval for which a barrier exists as shown in Fig. 2, where both the barrier height and “energy step” between the minima are plotted. In this range, and with χ near 1, the local minimum can move inside the domain [0,1], and an unstable fixed point (i.e., the location of the peak of the barrier) emerges, leading to two basins of attraction (Fig. 2). For α = β = 1 and χ = 0.9, this range is 1/10 < *s* < 5/18 . The requirement that χ, which characterizes the nonlinearity of the system, be near 1 implies that the phenomenon is nonperturbative. The value χ = 0 implies *R*_on_ = *R*_off_, which means that the network is composed of regular resistors. The asymptotic behavior of this single-element dynamical system is fully characterized by the simple basins of attraction of the potential and presents no exotic features. Typically, because one must have 0 ≤ *x* ≤ 1, the equations of motion of the single variables are supplied with (nonabsorbing) cutoff functions, e.g., $\frac{d}{d\tau }x=-W(x)f(x,y)$, with *W*(*x*) = 1 for {0 < *x* < 1} ∪ {*x* = 1, *f*(*x*, *y*) > 0} ∪ {*x* = 0, *f*(*x*, *y*) < 0} and zero otherwise.

When, instead of a single memory device, we have a circuit composed of many memristors, each with resistance *R*(*x_i_*) and with voltage generators *S_i_* in series (taken to be constant $S_{i}=\tilde{S}$), the differential Eq. 2 generalizes to a system of coupled and nonlinear ordinary differential equations. The network dynamics equation for the memory elements *x_i_*(*t*) is (*40*, *41*)

$$\frac{d}{\mathrm{dt}}\vec{x}=\frac{1}{\beta }{\left(I-\chi \Omega X\right)}^{-1}\Omega \vec{S}-\alpha \vec{x}$$ (4)

with $\chi =\frac{R_{\text{off}}-R_{\text{on}}}{R_{\text{off}}}<1$, and *X_ij_*(*t*) = *x_i_*(*t*)δ*_ij_*. The matrix Ω is the projection operator (Ω^2^ = Ω) on the vector space of cycles of 𝒢, the graph representing the circuit (*40*). In practice, Ω can be determined via the directed incidence matrix *B* of 𝒢 as Ω = *I* − *B*(*B^t^B*)^−1^*B^t^* (*31*, *42*–*44*). The fact that Ω is a projection operator is a mathematical representation of Kirchhoff’s circuit laws. Here, we choose *B* to be a random matrix to abstract the dynamical system from a particular circuit topology.

The system has a Lyapunov function when $\vec{S}$ is constant (see Supplementary Material A), and memristors are passive elements and thus cannot have positive Lyapunov exponents, e.g., the system cannot be unstable for long times (*24*). Passive components subject to DC voltage generators will approach an equilibrium, and thus, only transient forms of instability are possible. Exact information about the behavior of Eq. 4 has been difficult to obtain as the matrix inverse (*I* − χΩ*X*)^−1^ contains the variables *x_i_*(*t*) and is therefore hard to solve analytically. In addition, Bézout’s theorem for quadrics of order 2 with *N* variables suggests the number of equilibrium points (stable, unstable, or saddle) is exponential in the number of memristors (*45*), but at most, 2*^N^*. Intuitively, this can be seen considering that each memristor has two fixed points.

**Fig. 3.**
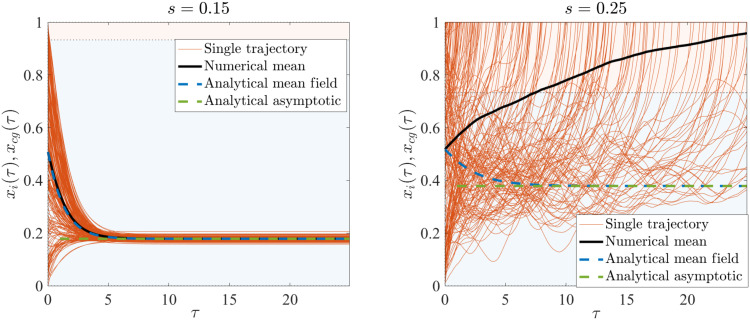
System trajectories in the laminar and transiently chaotic regimes. Dynamics of the system for low-voltage (*s* = 0.15) and high-voltage (*s* = 0.25) values. The mean field basins of attraction of the effective potential are represented by the background blue and red coloring. The initial condition for the elements of *x* is a uniform distribution in [0,1]. **Left:** Trajectories of the dynamical system for *s* = 0.15 and χ = 0.9 agree with the mean field description (equivalent to a single memristor). **Right**: Rumbling trajectories of the dynamical system for the case of the nonconvex potential, for *s* = 0.25, and in which the mean field equations fail to capture the dynamics of the network.

In the regime where the potential *V*(*x*, *s*) has multiple minima and the initial condition of the system lies outside of the attraction basin of the global minimum, the system exhibits instability. This can be seen in Fig. 3 (red curves), where for χ = 0.9, the system shows qualitatively different dynamics at different values of *s*. Here, we have assigned the initial conditions of the elements of $\vec{x}$ according to a uniform distribution on [0,1] and considered two cases for *s*: *s* = 0.15, for which the global minimum is located at *x_i_* ≈ 0.2, and *s* = 0.25, for which the global minimum is located at *x_i_* = 1 (see Fig. 2). The former case shows laminar behavior with the trajectories converging smoothly to *x_i_* ≈ 0.2. On the other hand, for *s* = 0.25, initial conditions for the elements of $\vec{x}$ are almost all within the local minimum’s basin of attraction because now the global minimum is at *x_i_* = 1; now, the system shows unstable dynamics, pushing trajectories to the other side of the barrier and converging to the global minimum *x_i_* = 1 (see Fig. 3).

**Fig. 4.**
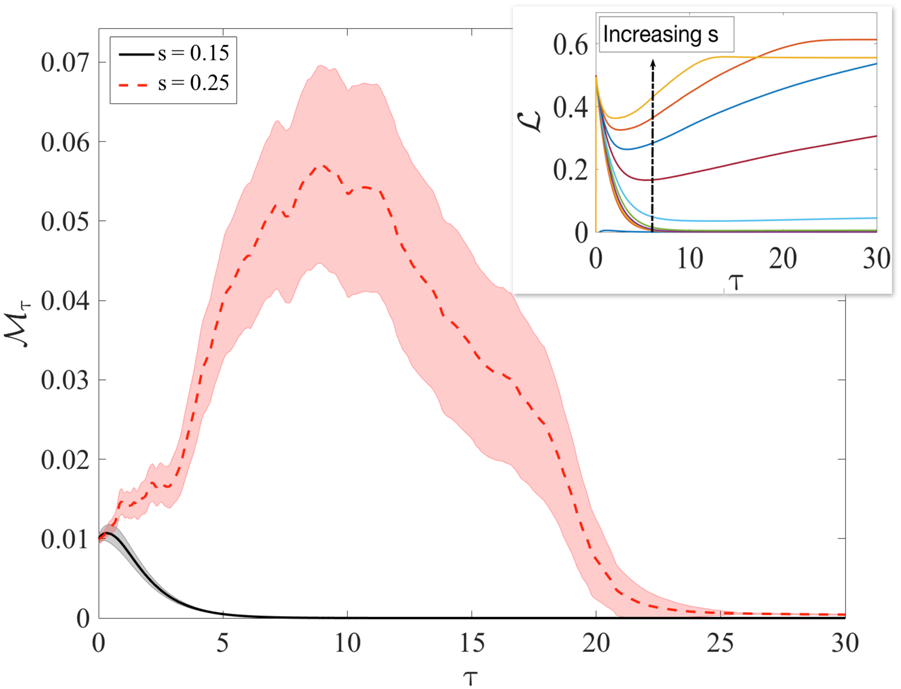
Laminar and transiently chaotic regimes. The growth and decay of the distance between perturbed trajectories *x*(τ) and *x*^ϵ^(τ) are shown as a function of time for *s* = 0.15 and *s* = 0.25 (shown for χ = 0.9). The distance is computed as ${ℳ}_{\tau }=N^{-1}{\left\|\vec{x}(\tau )-{\vec{x}}^{\epsilon }(\tau )\right\|}_{1}$. The perturbation ϵ on the initial condition is such that initially ℳ_0_ = 0.01. In the laminar regime (*s* = 0.15 shown in black), ℳ_τ_ quickly decays to zero, while it transiently grows before decaying in the unstable regime (*s* = 0.25 shown in red). To measure the growth and decay of *M*_τ_, we look at $\lambda =\frac{1}{\bar{\tau }}\text{log}\frac{{ℳ}_{\bar{\tau }}}{{ℳ}_{0}}$. For *s* = 0.15, we obtain λ = − 0.79(1), while for *s* = 0.25, we obtain λ = 0.2(1), and thus a positive local Lyapunov exponent. Inset: Evolution of the Lyapunov force as a function of time and for *s* ∈ [0.15,0.27]. Every curve is averaged over 100 initial conditions, and the shadow area represents the SD on the mean curves.

We quantify this behavior numerically using the distance between replicas of the system with similar initial conditions. Considering a central trajectory $\vec{x}(t)$, we generate others such that ${\vec{x}}^{\epsilon }(0)=\vec{x}(0)+\vec{\epsilon }$ where ${\left\|\vec{\epsilon }\right\|}_{1}=0.01N$, and where ‖ · ‖_1_ represents the vector 1-norm. It is natural to define the quantity ${ℳ}_{\tau }=N^{-1}{\left\|\vec{x}(\tau )-{\vec{x}}^{\epsilon }(\tau )\right\|}_{1}$, e.g., a normalized 1-norm that quantifies the deviation of the trajectories element by element and that is convenient because it is naturally normalized between 0 and 1. The results are shown in Fig. 4, where we see that for the case in which *s* = 0.15 (laminar), ℳ_τ_ decays to zero rapidly. For *s* = 0.25 (unstable), ℳ_τ_ grows for a transient approximately sevenfold longer before asymptotically reaching zero. For this reason, we dub this phenomenon as a rumbling transition because of the chaoticity of the individual trajectories, resulting from the nonlinearity of the interaction. The nonlinearity emerges from the inverse matrix (*I* − χΩ*X*)^−1^, which has its root in the essential nonlinearity of the memristive devices. Because of this, the transition from one minimum to the other is not laminar and is characterized by the instability of each single trajectory.

### Coarse-grained dynamics

We now make the intuitive picture above more precise by introducing an effective mean field potential. In a recent work, one of us has shown that the resolvent of large matrices exhibits statistical regularities, for which it can be approximated by an effectively one-dimensional matrix (*46*, *47*) that is universal at the zeroth order in the limit of weak correlations between the matrix elements.

If we define $\vec{f}=\Omega \vec{x}$ and the matrix

$$\tilde{A}=(\begin{array}{ccc} f_{1}(\vec{x}) & \cdots & f_{1}(\vec{x})\\ f_{2}(\vec{x}) & \cdots & f_{2}(\vec{x})\\ \vdots & \ddots & \vdots \\ f_{N}(\vec{x}) & \cdots & f_{N}(\vec{x}) \end{array})=\vec{f}⊗{\vec{1}}^{t}$$ (5)

we then have the approximate relation (details in Materials and Methods)

$${\left(I-\chi \Omega X\right)}^{-1}=I+\frac{1}{N}\frac{\chi }{1-\frac{1}{N}\chi \sum_{i=1}^{N}f_{i}(\vec{x})}\tilde{A}+O(\frac{1}{N})$$ (6)

Thus, if we define the operator mean as $\langle \vec{W}\rangle =N^{-1}\sum_{i=1}^{-1}W_{i}$ and the coarse-grained variable $x_{\text{cg}}=\langle \vec{f}(\vec{x})\rangle$, then we can write an effective one-dimensional dynamics

$$\begin{array}{c} \frac{d}{d\tau }x_{\text{cg}}=\frac{1}{\alpha \beta }(\langle \Omega \vec{S}\rangle +\frac{\chi \langle \Omega \vec{S}\rangle }{1-\chi x_{\text{cg}}}x_{\text{cg}})-x_{\text{cg}}+L(\vec{x})\\ =-\partial x_{\text{cg}}V(x_{\text{cg}},\chi )+L(\vec{x}) \end{array}$$ (7)

where $L(\vec{x})$ is an effective force due to the fact that the coarse graining is not exact. We see that at the zeroth order, *x_cg_* obeys dynamics similar to those of a single memristor, where, however, the parameter $\frac{s}{\alpha \beta }$ is replaced by the mean field value $s=\frac{\langle \Omega \vec{S}\rangle }{\alpha \beta }$. Thus, the equation above again represents gradient descent dynamics for a one-dimensional system, but where an effective external force $L(\vec{x})$ emerges from the interaction between the large set of (hidden) variables *x_i_* and which we can evaluate numerically on the dynamics. In a sense, while the *N* particles somehow feel a similar potential, these can interact as well, leading to a nontrivial collective dynamics. As a result, we find that the variable *x_cg_* is a natural dynamical order parameter to study for our system.

### Laminar versus transient unstable (rumbling) dynamics

We numerically examined the validity of the coarse-grained dynamics for various ranges of the parameters *s* and χ and found that it does fit the dynamics in some range of parameters. Specifically, the picture that emerges is represented in Fig. 3. In the laminar regime, the mean field dynamics accurately fits the dynamics of the system. This is always the case for χ ≪ 1, in which very few trajectories cross the barrier and the dynamics are therefore smooth. In this regime, the effective force ℒ(*x*) (which we call Lyapunov force for reasons that become clear soon) is typically small after a short transient, as we can see in the inset in Fig. 4. This implies that the equilibrium points of the dynamics, which are those satisfying $\frac{d}{d\tau }x_{\text{cg}}=0$, are well approximated by the mean field dynamics.

This gives the equilibrium value

$$x_{\text{cg}}^{*}=\frac{1}{N}\sum_{\text{ij}}\Omega_{\text{ij}}x_{j}^{*}=\frac{\alpha \beta -\sqrt{\alpha^{2}\beta^{2}-4\alpha \beta \chi \langle \Omega \vec{S}\rangle }}{2\alpha \beta \chi }$$ (8)

which matches simulations. This equation has many multiple possible solutions in high dimensions (see Supplementary Material B), explaining the large number of asymptotic states of the memristive dynamics commonly seen in numerical experiments (*48*). Yet, this also implies that the dynamics can be succinctly described by a scalar order parameter for the system and that the mean field and the effective potential do play a role.

The ability of the coarse-grained dynamics to capture the memristor network evolution changes abruptly when most *x_i_* have initial conditions outside of the attraction basin of the global minimum, as can be seen in Fig. 3 (right). Individual trajectories *x_i_*(*t*) now deviate from the coarse-grained value *x_cg_*(*t*) substantially, as we can see in Fig. 3 (right), with clear instabilities that lead them to the global equilibrium *x_i_*(*t*) = 1. While this might seem irksome at first, we argue that this phenomenon is worthy of attention. The effective Lyapunov force L(*x*) is now consistently pushing the system toward the mean field global minimum, suggesting that the mean field potential is still capturing important features of the asymptotic dynamics.

### Tunneling

To capture this effect as a tunneling phenomenon, we initialized the system around the local minimum using the initial conditions $\vec{x}(0)=x_{\text{lm}}(s)+\vec{\sigma }$, where *x*_lm_(*s*) is the location of the coarse-grained local minimum as depicted in Fig. 2, and $\vec{\sigma }$ is a random vector drawn from the Gaussian distribution 𝒩(0, σ). Given this, we performed Monte Carlo simulations and obtained the probability *P_t_* that the system hops from the local minimum to the global minimum or, in other words, the probability *P_t_* that the initial condition $\vec{x}(0)=x_{\text{lm}}(s)+\vec{\sigma }$ leads to $\vec{x}(t=\infty )=x_{\text{gm}}(s),$ where *x*_gm_(*s*) is the location of the global minimum as depicted in Fig. 2. We also note that *x*_lm_(*s*) shows an abrupt change for *s* = *s*_crit_ ≈ 0.206 as depicted in Fig. 2 as at that point, the local and global minima switch locations. This leads to two different effective tunneling directions for *s* > *s*_crit_ and *s* < *s*_crit_ (see the sketch in Fig. 1).

We first investigated how tunneling emerges as a collective behavior of the system.

**Fig. 5.**
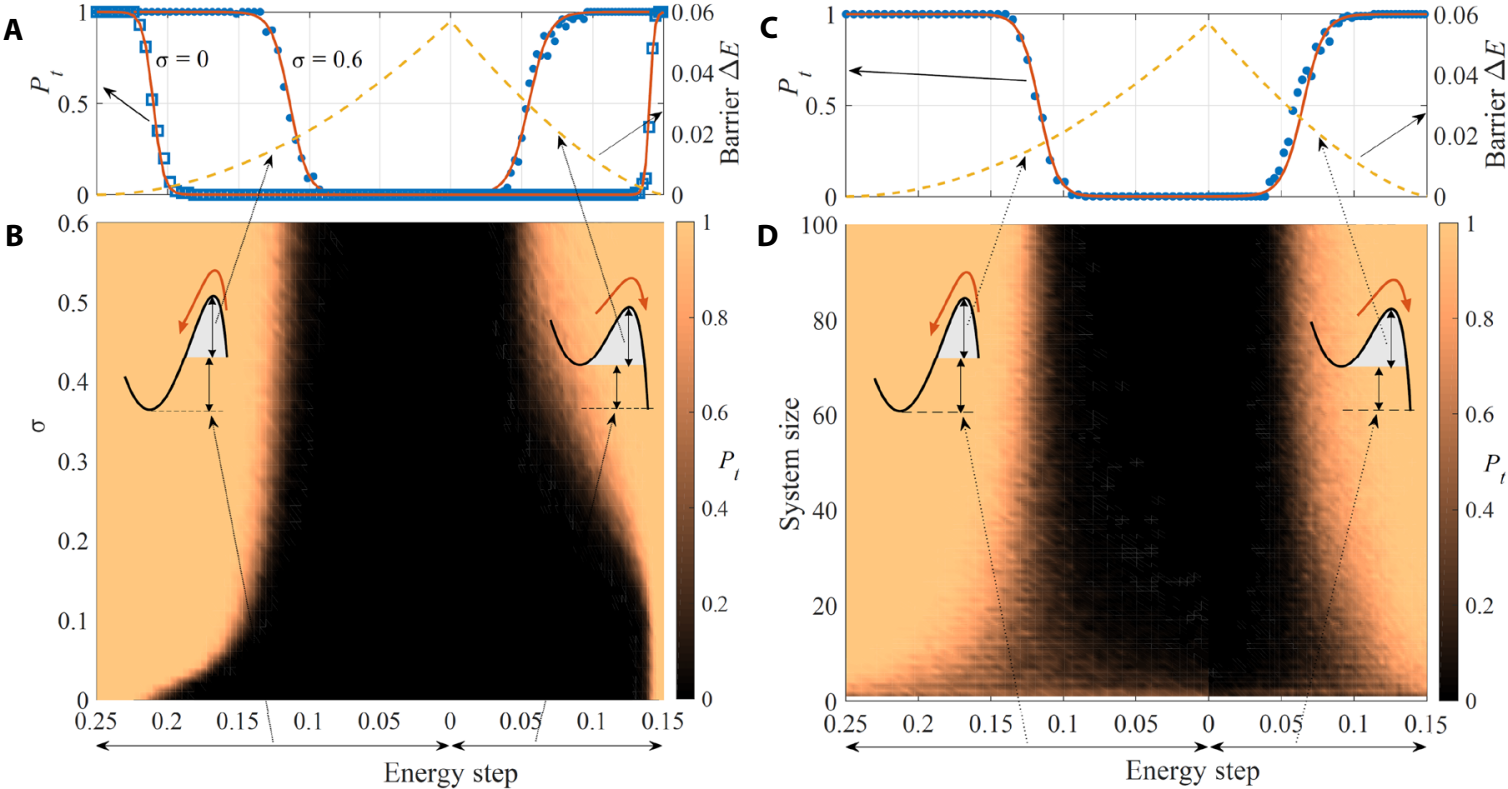
Tunneling probability *P_t_* from the local to the global minimum of *V(x,s)*. Insets (**A**) and (**B**) show *P_t_* evaluated by averaging over Gaussian initial conditions (σ = 0.5) centered on the local minimum of *V*(*x*, *s*). Inset (B) shows the probability *P_t_* as a function of the energy step between the two minima and the size of the system (or equivalently the number of memristors), while (A) shows a single section across (B) at a system of 100. Blue dots in (A) are the numerical evaluation of *P_t_*, while the solid red curve shows a sigmoidal fit. The dashed yellow curve is the barrier Δ*E* as a function of the energy step. Insets (**C**) and (**D**) are evaluated for system size = 100. Inset (D) shows *P_t_* as a function of the energy step between the two minima and the SD σ of the initial condition distribution. Inset (C) shows two sections of the plot in (D) for σ = 0 and 0.5. Blue dots and squares are the numerical evaluation of *P_t_*, while the solid red curves are sigmoidal fits. The dashed yellow curve is the barrier Δ*E* as a function of the energy step.

Figure 5B reports the tunneling probability for σ = 0.5, for system sizes from 1 to 100 components, and for the entire range of *s*. We observe that at small system size, the tunneling probability is proportional to the number of initial conditions that fall into the global minimum attraction basin. However, at increasing size, the probability shifts into an almost perfect sigmoid as a function of the energy barrier Δ*E* (see also Fig. 5B). This transition is smooth and qualitatively equivalent for both tunneling directions. Therefore, the system shows a size-dependent transition from gradient dynamics to a collective tunneling toward the global optimum, led by the emergent Lyapunov force.

We also investigated the dependence of *P_t_* on σ, the spread of the initial conditions. Figure 5D reports the tunneling probability *P_t_* as a function of σ for a system of 50 components. An interesting feature manifests itself: For σ = 0, there is a nonnull height of the barrier Δ*E* for each tunneling direction below which the system is still able to tunnel toward the global minimum with probability 1 (this is highlighted in Fig. 5C). This shows that the Lyapunov force is an intrinsic collective large system–size feature present even in the absence of any randomness in the initial conditions.

On the basis of this, we investigated whether transient instability could be attributed to a local amplification of perturbations due to nonnormality of the Jacobian (*49*). We analyzed a large number of local minima via Monte Carlo (see Supplementary Material C), finding that the nonnormality of the Jacobian does not lead to any amplification phenomenon. This also leaves us with the only option that the instability of Fig. 4 is due to a nonperturbative and cooperative phenomenon between the dynamical variables. We have also analyzed random initial conditions, which span the interval [0,1]. The picture that emerges is also qualitatively and quantitatively similar to the one presented above (for details, see Supplementary Material D).

**Fig. 6.**
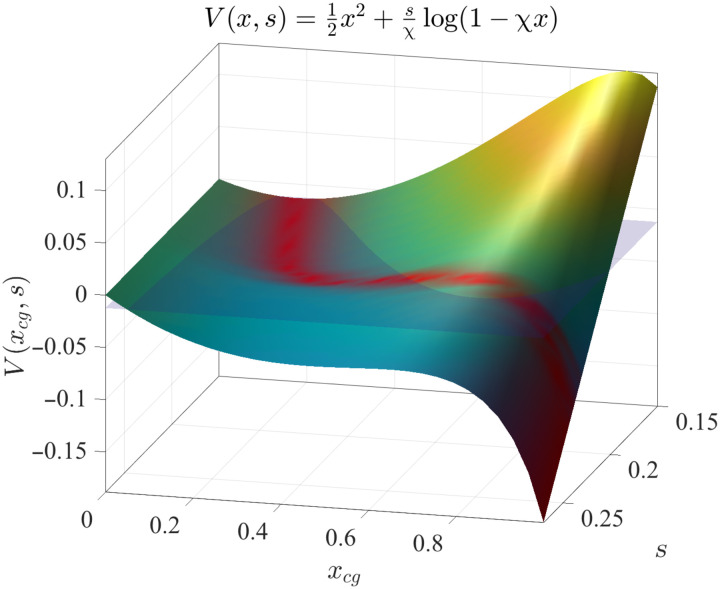
Asymptotic states as a function of the voltage *s* in the effective potential. We plot the average asymptotic state $E[\langle \vec{x}(t=\infty )\rangle ]$ (red curve) of the memristor coarse-grained variable, obtained from 400 Monte Carlo instances for each point $\left(s,\vec{x}\right)$, on a grid of 50 points *s* ∈ [0.15,0.27] and 30 points *x_i_* ∈ [0,1] and for χ = 0.9, obtained with a Euler integration method with *dt* = 0.1, and tested against a Runge-Kutta fourth-order integration method for stability. The transparent plane is for visual aid to show that the average climbs the barrier.

The system displays this behavior even when randomly initialized and was investigated systematically. To highlight the role of the basin of attraction of the global minimum, we used Monte Carlo simulations, randomizing over the initial conditions on [0,1]. The results are shown in Fig. 6. The surface shows the potential *V*(*x*, *s*) as a function of *x_cg_* and *s*. The superimposed red curve represents the asymptotic average position $E[\langle \vec{x}(t=\infty )\rangle ]$, where *E*[·] is the average over the Monte Carlo samplings and $\langle \vec{x}\rangle =\frac{1}{N}\sum_{i}x_{i}$ is the average over the components. As can be seen, before the barrier disappears, the dynamics reaches the right minimum of the potential *V*(*x*, *s*) with probability one. A glimpse of the structure of the basins of attraction can be obtained by fixing the initial conditions homogeneously for all but two variables *x_i_* and looking at the resulting asymptotic state in these two variables (see the Supplementary Material D for details) near the transition point.

## DISCUSSION

We have presented a novel mechanism in which barrier escapes emerge in the effective description of a classical many-body system, and provided a qualitative and quantitative analyses of this picture in a well-defined model. We presented a class of dynamical systems, derived from networks of memristors, that can be mapped to an effective one-dimensional dynamical system. The resulting dynamics is characterized by a potential that depends on the external applied voltage and an effective force that occurs when the system becomes Lyapunov unstable. The result of this instability is that the effective representation may jump between basins of attraction, even through a barrier.

This transition is due to the large number of directions in which the system can travel, via a sequence of saddle points in the dynamics, and from which the local instability emerges. This is compatible with previous investigations of memristive circuit dynamics applied to self-organizing logic gates (*50*, *51*) and the observation of instanton-like behavior in the dynamics (*52*, *53*).

Our analysis of the effective Lyapunov force provides an explanation of the observed tunneling as an epiphenomenon of the interaction between the memory elements. In principle, this might fit into a coarse-graining argument of the hidden variables (*54*), which will be considered in the future. This said, we wish to stress that the system we studied is a-thermal, i.e., no stochastic forces were introduced in our analysis, and that the effective Lyapunov force is far from random. The results presented in the present paper have been also extended to other types of more memristor dynamics, incorporating both window functions and nonlinear currents functions, confirming that the “tunneling” picture is present also in more realistic component descriptions (see Supplementary Material E).

The main message of this paper is that the introduction of hidden variables in a dynamical system can lead to transitions between local and global minima in the effective description via instabilities in the full system. This occurs also in classical interpretations of quantum mechanics, for instance, in Bohm’s mechanics (*4*) and in attempts of generalizing gradient descent (*55*). While a similar behavior has been observed in memristor-enhanced simulated annealing (*23*), this has never been properly understood or characterized analytically.

An issue of immediate importance is whether this behavior can be generalized to arbitrary dynamical systems, i.e., whether we can embed any set of coupled ordinary differential equation (ODE) in a larger dynamical space. Among these, of particular interest are dynamical equations based on the gradient of potentials, which have applications in optimization. In this case, the interesting question is how minima, maxima, and saddle points are mapped in the new dynamical system. All the questions above will be investigated in future papers.

Nonetheless, we hope that our paper can spark further interest in the study of tunneling in dynamical systems via “hidden variables,” with their interesting properties and with important applications to computer science and optimization in particular (*23*, *29*, *52*, *53*) or machine learning, where similar phenomena have been previously observed (*56*, *55*).

From the point of view of experiments in neuromorphic systems, our paper predicts that with the increase in applied voltage in networks of interconnected memristors, system-level switching should be observed. Within the context of nanowire network, a similar system switching has been recently observed both experimentally and numerically in (*57*). In particular, it has been observed that in silver nanowires below a threshold voltage *V*_th_, the system slowly relaxes toward an asymptotic resistive state. Above the threshold voltage *V*_th_, the system experiences a marked shift in behavior, in which the resistivity of the material changes by three order of magnitudes. As exciting as these results are, however, an interpretation for the switching in terms of an energy function has not been provided. The present paper shows, at least within the context of the models we studied, that the dynamical switching can be interpreted as a symmetry breaking phenomenon. The interpretation we provide here is that the shift is due to the change of the effective potential in the system, which develops a new minimum as the voltage increases and then becomes predominant and leads to a first-order transition. As we have seen in this paper, also in the experimental results of (*57*), a chaotic transition is observed. In this sense, this paper provides a possible explanation for the complex phenomenology observed in silver nanowires. We hope that more experiments will further unveil the properties of such dynamical transition and the deep connection between neuromorphic systems and statistical physics.

## MATERIALS AND METHODS

### Numerical simulations

The numerical results in this paper were obtained using a Euler integration scheme with a step size *dt* = 0.1. To test the validity of this scheme, we have also tested that the same phenomenology emerges to smaller values of *dt* (*dt* = 0.01 and *dt* = 0.001). Moreover, we have performed independent simulations using a fourth order Runge-Kutta method with similar step sizes, obtaining comparable results both in terms of the general phenomenology and the transient Lyapunov exponents.

### Large-*N* resolvent and mean field equations

A resolvent is a matrix of the form

$$R(z;A)={\left(\mathrm{zI}-A\right)}^{-1}$$ (9)

It is not hard to see that in Eq. 4, we have a similar matrix inverse, which is, unfortunately, a technically difficult task in general, both numerically and analytically. For this purpose, we now introduce an approximate resolvent based on the notion that the matrix *A* is random. In particular, we discuss the theorem used in the main text to derive the mean field potential. Such theorem has been derived with the intention of studying resolvent matrices, but we will see below how to apply it to the memristor dynamics.

#### Theorem

Let *A* = (*a_ij_*) be a random *N* × *N* matrix, characterized by the joint probability density function of the entries *P_A_*(*a*_11_, …, *a_NN_*). Let *a_ij_* ≥ 0, ρ(*A*) < 1, β > 1, and

$${\langle a_{\text{ij}}\rangle }_{A}=z_{i}/N>0$$ (10)

$${\langle a_{\text{ij}}a_{k\ell }\rangle }_{A}-{\langle a_{\text{ij}}\rangle }_{A}{\langle a_{k\ell }\rangle }_{A}∼\frac{C_{\text{ij}}}{N^{\beta }}\delta_{\mathrm{ik}}\delta_{j\ell }\;\text{as}\;N\to \infty$$ (11)

for all *i*, *j*, with 〈(·)〉*_A_* defined as

$${\langle (\cdot )\rangle }_{A}=\int da_{11}\cdots da_{\mathrm{NN}}P_{A}(a_{11},\ldots ,a_{\mathrm{NN}})(\cdot )$$ (12)

and *C_ij_* be constants. Then, we have

$${\left(I-A\right)}^{-1}=I+\frac{1}{N}\frac{1}{1-\bar{z}}\vec{z}⊗{\vec{1}}^{t}+O(\frac{1}{N^{\gamma }})$$ (13)

where $\bar{z}=(1/N)\sum_{i=1}^{N}z_{i}$, and $z_{i}=\sum_{j=1}^{N}A_{\text{ij}}$, with γ > 0. The results above hold irrespective of the precise form of *P_A_*. For a proof, see (*46*), and an application of the formula above to complex datasets is given in (*47*). The theorem above applies to random matrices with certain sum constraints. While the theorem is stated for a particular form of the correlation between the elements, as a matter of fact, it is more general, and it applies to matrix functions whose element correlations are sufficiently weak. Nonetheless, for finite *N*, the expansion has corrections that are important and lead to the Lyapunov force, which we estimated numerically in the main text. The Lyapunov force can be interpreted simply as the deviation from the mean field theory.

In our case, the ensemble *A* is the one of the random projector operators Ω multiplying the state variable matrix *X*. We will make hereby the connection between the theorem and our setup. The formula for the resolvent is exact when the span of the operator Ω is one-dimensional, as it can be promptly seen using the Sherman-Morrison formula.

We now consider the equation

$$\frac{d}{\mathrm{dt}}\vec{x}=\frac{1}{\beta }{\left(I-\chi \Omega X\right)}^{-1}\Omega \vec{S}-\alpha \vec{x}$$ (14)

The technical condition lim_*N* → ∞_*N*〈*a_ij_*〉*_A_* = *c_ij_* applies in the case of memristive dynamics if we consider the fact that Ω is a projector operator. As shown in (*41*), typically, $\Omega_{\text{ij}}\approx \frac{c}{N}$ because of the condition Ω^2^ = Ω. As such, if we identify *A* = χΩ*X*, because we impose 0 < *x_i_* < 1 dynamically, and χ < 1, then we have that $a_{\text{ij}}<\frac{\chi |c|}{N}$, which satisfies the technical condition. The numerical results are obtained for a number of memristors equal to *N* = 200, and we chose for the numerical analysis ∣Span(Ω)∣ = *N* − *M*, with *M* = 50. For random matrices, we generate Ω = *A^t^*(*AA^t^*)^−1^*A*, with *A* being random matrix of size *N* × *M*, and *a_ij_* being randomly distributed in [0,1].

We now discuss how to apply the result above to the dynamics of memristors. Let us define $f_{i}(\vec{x})=\sum_{j}\Omega_{\text{ij}}x_{j}$. It is not hard to see from the definition above that $z_{i}=\chi f_{i}(\vec{x})$. Let us define $\vec{f}=\{f_{i}(\vec{x})\}$. We then have

$$\tilde{A}=(\begin{array}{ccc} f_{1}(\vec{x}) & \cdots & f_{1}(\vec{x})\\ f_{2}(\vec{x}) & \cdots & f_{2}(\vec{x})\\ \vdots & \ddots & \vdots \\ f_{N}(\vec{x}) & \cdots & f_{N}(\vec{x}) \end{array})=\vec{f}⊗{\vec{1}}^{t}$$ (15)

The expansion above can be rewritten as

$${\left(I-\chi \Omega X\right)}^{-1}=I+\frac{1}{N}\frac{\chi }{1-\chi \frac{1}{N}\sum_{i=1}^{N}f_{i}(\vec{x})}\vec{f}⊗{\vec{1}}^{t}+O(\frac{1}{N^{\gamma }})$$ (16)

from which we obtain, if we call $\Omega \vec{S}=\Omega \vec{S}$

$$\frac{d}{\mathrm{dt}}\vec{x}=\frac{1}{\beta }(\Omega \vec{S}+\frac{\chi \frac{1}{N}\Omega \vec{S}\cdot \vec{1}}{1-\chi \frac{1}{N}\vec{f}\cdot \vec{1}}\vec{f})-\alpha \vec{x}+O(\frac{1}{N^{\gamma }})$$ (17)

Now, we have the identity $\Omega \vec{f}=\vec{f}$ and $\Omega \vec{S}=s$. Thus, if we project the equation using Ω, we have

$$\frac{d}{\mathrm{dt}}\vec{f}=\frac{1}{\beta }(\Omega \vec{S}+\frac{\chi \frac{1}{N}\Omega \vec{S}\cdot \vec{1}}{1-\chi \frac{1}{N}\vec{f}\cdot \vec{1}}\vec{f})-\alpha \vec{f}+O(\frac{1}{N^{\gamma }})$$ (18)

The asymptotic states are thus determined by the equation

$$\frac{1}{\beta }(\Omega \vec{S}+\frac{\chi \frac{1}{N}\Omega \vec{S}\cdot \vec{1}}{1-\chi \frac{1}{N}\vec{f}\cdot \vec{1}}\vec{f})-\alpha \vec{f}=0$$ (19)

or

$$\vec{f}=\Omega \vec{x}=\frac{1}{1-\frac{\chi }{\alpha \beta }\frac{<\Omega \vec{S}>}{1-\chi x_{\text{cg}}}}\frac{\Omega \vec{S}}{\alpha \beta }$$ (20)

where we defined $\langle \vec{G}\rangle =\frac{1}{N}\vec{G}\cdot \vec{1}$. The asymptotic states can be obtained up to a linear transformation ${\vec{x}}^{*}={\vec{x}}^{*}+(I-\Omega )\vec{r}$ for an arbitrary $\vec{r}$. Such freedom is exactly rooted in the Kirchhoff laws for the circuit (*31*). The equation above, however, cannot be solved without first finding a value for *x_cg_*. The differential equation for $x_{\text{cg}}=\frac{1}{N}\vec{f}\cdot \vec{1}$ can be obtained by summing over all the indices on both sides of Eq. 18 and dividing by *N*, obtaining

$$\partial_{t}x_{\text{cg}}=\frac{1}{\beta }(\langle \Omega \vec{S}\rangle +\frac{\chi \langle \Omega \vec{S}\rangle }{1-\chi x_{\text{cg}}}x_{\text{cg}})-\alpha x_{\text{cg}}+L(\vec{x})$$ (21)

where $L(\vec{x})$ is an effective force we will discuss in a moment, due to the rest *O*(1/*N*) in Eq. 18. In principle, we could also use a double-constrained version of Eq. 13 for the resolvent, obtained in (*46*), but we find empirically that the mean field Eq. 21 provides a good estimate of the dynamics in the laminar regime.

The equilibrium points are obtained by the solution of

$$\frac{1}{\beta }(\langle \Omega \vec{S}\rangle +\frac{\chi \langle \Omega \vec{S}\rangle }{1-\chi x_{\text{cg}}}x_{\text{cg}})-\alpha x_{\text{cg}}=0$$ (22)

which is equivalent to a mean field equation of the form

$$x_{\text{cg}}=\frac{\langle \Omega \vec{S}\rangle }{\alpha \beta }(1+\frac{\chi x_{\text{cg}}}{1-\chi x_{\text{cg}}})$$ (23)

The solutions are given by

$$x_{\text{cg}}^{*}=\frac{1}{N}\sum_{\text{ij}}\Omega_{\text{ij}}x_{j}^{*}=\frac{\alpha \beta -\sqrt{\alpha^{2}\beta^{2}-4\alpha \beta \chi \langle \Omega \vec{S}\rangle }}{2\alpha \beta \chi }$$ (24)

By setting $\frac{\langle \Omega \vec{S}\rangle }{\alpha \beta }=s$, we obtain a mapping to the one-dimensional case. Let us now discuss the emergence of the lack of convexity of the potential *V*(*x*, *s*) as a function of *s*; specifically, let us analyze the situation in which the potential has a minimum or a maximum. The coarse-grained variable is given, as mentioned earlier, by $x_{\text{cg}}=\frac{1}{N}\sum_{\text{ij}}\Omega_{\text{ij}}x_{j}$. First, we focus on the potential on a compact support *D* = [*x*_min_, *x*_max_], determined via the condition $x_{\text{min}}=\text{mi}n_{\vec{x}\in {\left[0,1\right]}^{N}}(x_{\text{cg}})$ and $x_{\text{max}}=\text{ma}x_{\vec{x}\in {\left[0,1\right]}^{N}}(x_{\text{cg}})$. For *s* < 0, the potential has only a local minimum (which is also an absolute minimum) at *x_cg_* = max (0, *x*_min_). For *s* > 0, the potential $V(x,s)=\frac{1}{2}x^{2}+\frac{s}{\chi }\text{log}\;(1-\chi x)$ can have, at most, two points in which its derivative in *x* is zero, given by the values $\frac{x^{+}=1\pm \sqrt{1-4s\chi }}{2\chi }$. While *x*^−^ is always a local minimum, *x*^+^ is always a local maximum of the potential if two minima are present. However, there is an intermediate set of values, given by $s=[0,\frac{1}{4\chi }]$, in which the potential has only a single minimum in the domain [*x*_min_, *x*_max_], as *x*^+^ is outside the domain. In this case, the local minimum *x*^−^ is also an absolute minimum in the domain [0,1]. However, if χ is close enough to 1 and *s* large enough, we have that *x*^+^ moves inside the domain, and thus, the potential has two local minima in [0,1]: one at *x*^−^ and one at *x*_max_. If *s* increases further, we can have that *V*(*x*_max_) < *V*(*x*^−^), implying that the potential is not nonconvex, and a barrier of height Δ*E*(*s*) = *V_s_*(*x*^+^, χ) − *V_s_*(*x*^−^, χ) emerges between the local minimum *x*^−^ and the absolute minimum at *x*_*x*max_.

## Supplementary Materials

### This PDF file includes:

A: Proof of Lyapunov function for sharp boundaries B: Projected Dynamics and equilibrium points C: Local stability analysis and pseudospectral triviality D: Basins of attraction E: Generalization to other window functions and more physically realistic models Figs. S1 to S5
