# Supplementary material for: Global minimization via classical tunneling assisted by collective force field formation
Source: Sci Adv. 2021 Dec 22;7(52):eabh1542. doi: 10.1126/sciadv.abh1542 (PMC8694608; doi:10.1126/sciadv.abh1542)
Supplement: Supplementary file 1 — A: Proof of Lyapunov function for sharp boundaries B: Projected Dynamics and equilibrium points C: Local stability analysis and pseudospectral triviality D: Basins of attraction E: Generalization to other window functions and more physically realistic models Figs. S1 to S5 [file sciadv.abh1542_sm.pdf]

Science Advances
AAAS

Supplementary Materials for

Global minimization via classical tunneling assisted by collective force field formation

Francesco Caravelli*, Forrest C. Sheldon, Fabio L. Traversa

*Corresponding author. Email: caravelli@lanl.gov

Published 22 December 2021, *Sci. Adv.* **7**, eabh1542 (2021)
DOI: 10.1126/sciadv.abh1542

This PDF file includes:

A: Proof of Lyapunov function for sharp boundaries
B: Projected Dynamics and equilibrium points
C: Local stability analysis and pseudospectral triviality
D: Basins of attraction
E: Generalization to other window functions and more physically realistic models
Figs. S1 to S5

A: Proof of Lyapunov function for sharp boundaries

In this section we provide a proof of the fact that the system is dissipative when each state variable is far from the boundary $x_i = 1$ [47,50]. We consider the case of infinitely sharp boundaries, e.g. $\lim_{p\rightarrow\infty} W_p(x) = \theta(x)\theta(1-x)$. In order to prove it, we attempt to write a Lyapunov function of the form for the differential equation (when boundaries are not considered)

$$\frac{d}{dt}\vec{x} = \frac{1}{\beta}(I-\chi\Omega X)^{-1}\Omega\vec{S} - \alpha\vec{x}, \quad (1)$$

which we can rewrite as

$$\frac{1}{\alpha}(I-\chi\Omega X)\frac{d}{dt}\vec{x} = \frac{1}{\alpha\beta}\vec{s} - (I-\chi\Omega X)\vec{x}, \quad (2)$$

where we wrote $\Omega\vec{S} = \vec{s}$.

$$L = \frac{1}{3}\vec{x}^T X\vec{x} - \frac{\chi}{4}\vec{x}^T X\Omega X\vec{x} - \frac{1}{2\alpha\beta}\vec{x}^T X\vec{s}. \quad (3)$$

After taking a time derivative of the Lyapunov function above, we get

$$\begin{aligned}
\frac{dL}{dt} &= \dot{\vec{x}}^T\left(X\vec{x} - \chi X\Omega X\vec{x} - \frac{1}{\alpha\beta}X\vec{s}\right) = \dot{\vec{x}}^T X\left(\vec{x} - \chi\Omega X\vec{x} - \frac{1}{\alpha\beta}\vec{s}\right) \\
&= -\dot{\vec{x}}^T X\left(\frac{1}{\alpha\beta}\vec{s} - (I-\chi\Omega X)\vec{x}\right) \text{ which is Eqn. (2)} \\
&= -\frac{1}{\alpha}\dot{\vec{x}}^T X(I-\chi\Omega X)\dot{\vec{x}} = -\frac{1}{\alpha}\dot{\vec{x}}^T(X-\chi X\Omega X)\dot{\vec{x}} \\
&= -\frac{1}{\alpha}\dot{\vec{x}}^T\sqrt{X}(I-\chi\sqrt{X}\Omega\sqrt{X})\sqrt{X}\dot{\vec{x}} = -\frac{1}{\alpha}||\sqrt{X}\dot{\vec{x}}||^2_{(I-\chi\sqrt{X}\Omega\sqrt{X})}
\end{aligned} \quad (4)$$

We obtain that if $I-\chi\sqrt{X}\Omega\sqrt{X} \succ 0$ (and $X \neq 0$), the Lyapunov function has negative derivative always. This occurs if $x_i \in [0,1]$, which is a key factor in the dynamics, it is not hard to see that $\sqrt{X}\Omega\sqrt{X} \prec 1$ (if $x_i \in [0,1]$), and since $\chi < 1$, the Lyapunov property applies also in this case. The existance of a Lyapunov function also implies that there is a global notion of "energy" beyond the mean-field potential provided in the main text.

B: Projected Dynamics and equilibrium points

One of the advantages of using an analytical framework to study memristor dynamics is that many subtleties of nonlinear circuits become explicit in this setup. In this regard, it is interesting to recall the fact that $\Omega^2 = \Omega$. As mentioned earlier, the fact that this matrix is a projector operator is essentially a consequence of the Kirchhoff' laws. Such freedom implies a redundancy in the way in which we can effectively represent the dynamics, as investigated in earlier papers on the subject [42,43,44]. In fact, in order to obtain the matrix Ω from the circuit, one has the liberty of choosing a particular reference spanning tree, whose details are clarified in [33]. Such freedom is not hidden, but is evident from the fact that if we perform the transformation $\vec{S}' = \vec{S} + (I-\Omega)\vec{k}$ for an arbitrary vector $\vec{k}$, the dynamics is unchanged (this is reminiscent of the "toy" gauge freedom which is typically present in circuits). We now see that such freedom is also present in the representation of the fixed points. We can in fact write $\vec{x}_j' = \vec{x}_j + (I-\Omega)\vec{k}$, without effectively changing the coarse grained variable fixed point state.

Let us consider the two formulations of the dynamics, based on the fact that $\vec{x} = \Omega\vec{x} + (I-\Omega)\vec{x} = \vec{x}_c + \vec{x}_e$, in which $\vec{x}_c \cdot \vec{x}_e = 0$ because Ω is an orthogonal projector.

Given the dynamics introduced in the bulk of the paper, we have

$$\frac{d\vec{x}}{dt} = \frac{1}{\beta}(I-\chi\Omega X)^{-1}\Omega\vec{S} - \alpha\vec{x}, \tag{5}$$

from which, projecting via Ω and $\Omega_B = I-\Omega$, we have, using the series expansion for the matrix expansion, that

$$\begin{aligned} \frac{d\vec{x}_c}{dt} &= \frac{1}{\beta}(I-\chi\Omega(X_e+X_c))^{-1}\Omega\vec{S} - \alpha\vec{x}_c, \\ \frac{d\vec{x}_e}{dt} &= -\alpha\vec{x}_e. \end{aligned} \tag{6}$$

It follows that since $\vec{x}_e$ falls exponentially to zero, the fixed points are those such that $\vec{x}_e = 0$. Given $\vec{x}_c = \vec{f}$, the basin of attraction for the fixed points $\vec{f}^*$ is up to an arbitrary vector $\vec{x}_e = (I-\Omega)\vec{k}$ added to the initial conditions. For the asymptotic fixed points emerging from the mean field dynamics, this implies that, since x^*_{cg} must be such that $\frac{1}{N}\sum_{ij}\Omega_{ij}x^*_j = a$, then necessarily $\sum_j \Omega_{ij}x^*_j = x^*_i$. Thus, if Ω_{ij} has a span of $N-M$ vectors, $\vec{x}^* = \sum_{k=1}^{M-N} a_k\vec{n}_k$, where $\vec{n}_k$'s are the basis vectors of Span(Ω). It follows that the solutions of the problem are such that, if we call $\tilde{n}_k = \sum_i(\vec{n}_k)_i$, the solutions are of the form $\sum_k a_k\tilde{n}_k = a$.

C: Local stability analysis and pseudospectral triviality

For the single memristor dynamical system, the stability of the equilibrium is determined by the condition $-\partial_x^2 V(x^*) > 0$, with $\partial_x V(x) = 0$. This is equivalent to the condition

$$\frac{2\alpha\beta\left(-\alpha\beta + \sqrt{\alpha\beta(\alpha\beta-4s\chi)} + 4s\chi\right)}{\left(\sqrt{\alpha\beta(\alpha\beta-4s\chi)} - \alpha\beta\right)^2} < 0. \tag{7}$$

For reference, in the main text we consider the values $\alpha = \beta = 1$ and $\chi = 0.9$, and the dynamical instability occurs around $s \approx 0.27777(8)$.

The dynamics of our system can be described by the differential equations:

$$\frac{d\vec{x}}{dt} = \vec{f}(\vec{x}) \tag{8}$$

where $\vec{f} = [f_1, f_2 \cdots, f_S]^T$ is a set of nonlinear functions.

Since we are interested in our system escaping from the equilibrium point, we study numerically the equilibria $\vec{x}*$ which must satisfy

$$\vec{x}(\vec{x}^*) = 0. \tag{9}$$

Since the system can have an exponential number of fixed points, such search cannot be exaustive. However, finding these points is relatively easy and can be done numerically by initializing the dynamics randomly and letting the system evolve into one of these minima.

The local dynamics near each equilibrium point is described by the Jacobian matrix evaluated at an equilibrium point. An equilibrium point is stable if under any infinitesimally small perturbation, $\Delta\vec{x}(0)$, eventually decays to zero, i.e., $\lim_{t\to\infty}\Delta\vec{x}(t) = 0$. In the vicinity of an equilibrium point, the time evolution of a perturbation can be written as

$$\Delta\vec{x}(t) = e^{DJt}\Delta\vec{x}(0). \tag{10}$$

Therefore, the spectrum of DJ is relevant for local stability analysis. If $\Lambda($ M$)$ is the set of eigenvalues of $\mathbf{M}$, then the equilibrium point is stable if all eigenvalues have negative real part. For stability near the boundaries, the dynamical system is given by

$$\frac{d}{dt}x_j = W_j(x_j)f_j(\vec{x}) \tag{11}$$

where $W_p(\vec{x})$ is a window function enforcing $0 \le x \le 1$ and $f_j(\vec{x}) = (\frac{1}{\beta}(I-\chi\Omega X)^{-1}\Omega\vec{S} - \alpha\vec{x})_j$. In the following we consider the non-absorbing window function $W(x_i) = \theta(-f_i)\theta(x_i) + \theta(f_i)\theta(1-x_i)$, where we relaxed to $\theta(a)$ to $\theta_p(a) = \frac{1}{2} + \frac{1}{2}\tanh(pa)$, for numerical stability [9]. The numerical results shown in the paper are obtained for

$p = 100$, which is a good approximation of the Heaviside theta function $\theta(x)$. The full Jacobian of the dynamics is given by

$$DJ^f_{ij} = \partial_{x_i}(W_j(x_j)f_j(\vec{x})) = W_j(x_j)\partial_{x_i}(f_j(\vec{x})) + f_j(\vec{x})\partial_{x_i}W_j(x_j). \tag{12}$$

The Jacobian for the dynamical system without window functions can be evaluated exactly, and is given by (using $\partial_\alpha A^{-1} = -A^{-1}(\partial_\alpha A)A^{-1}$) by (δ_{ij}) is the Kronecker delta):

$$\begin{aligned} DJ_{ji} &= \partial_{x_i} f_j(\vec{x}) \\ &= \frac{\chi}{\beta\alpha}\sum_{krpm}(I-\chi\Omega X)^{-1}_{jk}\Omega_{kr}\delta_{ri}(I-\chi\Omega X)^{-1}_{rm}(\Omega\vec{S})_m - \delta_{ji} \\ &= \frac{\chi}{\alpha\beta}P_{ji}V_i - \delta_{ji}, \end{aligned} \tag{13}$$

where $P = (I-\chi\Omega X)^{-1}\Omega$ and $\vec{V} = P\vec{S}$, and which we could use to evaluate the Local Lyapunov Exponents or study local stability. The local flow divergence is obtained via the relation

$$\delta\vec{x}(t_{n+1}) = \big(I + DJ^f(t_n)dt\big)\delta\vec{x}(t_n). \tag{14}$$

The spectrum of the Jacobian at the fixed point has been studied first in [44], showing that the spectrum is real even if the matrix is not symmetric. Despite this, non-normal Jacobian matrices can in principle exhibit amplification of perturbations on a stable equilibrium and it is worth of careful scrutiny in this paper. Small perturbations of a stable equilibria typically decay in exponential time. In the case of non-normal matrices however, even if the eigenvalues have all real part negative, can exhibit a transient instability. How non-normal a matrix M is quantified by (any) norm of the matrix $N = (M^tM - MM^t)/2$. The Jacobian matrix of our system can be written in the form $DJ(X) = P^2\mathscr{S}$, where $P = (I-\chi\Omega X)^{-1}\Omega$ and $\mathscr{S}_{ij} = S_i\delta_{ij}$. It follows that $N = \frac{P^2\mathscr{S} - \mathscr{S}(P^t)^2}{2}$. This is not inconsistent with the notion of stability. As perturbations increase in magnitude via a transient phase, the effect on eigenvalues is more pronounced. For long times, if the system is linear, the long term dynamics is still dominated by the negativity of the real part of the spectrum. However, in the case of non-normal matrices, the transient dynamics can be no longer consistent with the traditional picture of asymptotic stability.

For this reason we go beyond a simple spectral analysis. Generalizations of the notion of eigenvalues have been studied in the literature. In general, the eigenvalues of DJ can be defined: $\Lambda(DJ) = \{z \in \mathbb{C} : \det(zI - DJ) = 0\}$, meaning that if z is an eigenvalue of DJ then by convention the norm of $(zI-DJ)^{-1}$ is defined to be infinity [51]. But if $||(zI-DJ)^{-1}||$ is finite and very large, as is often the case with perturbed non-normal matrices, then the pseudospectrum of DJ must be considered. The 'ε-pseudospectrum' is a generalization of the notion of eigenvalues which depend on a real parameter ε, and can be defined in various equivalent ways [51]. We use the following definition:

$$\Lambda_\varepsilon(DJ) = \{z \in \mathbb{C} : ||(zI-DJ)^{-1}|| \geq \varepsilon^{-1}\}. \tag{15}$$

If a matrix is normal then its ε-pseudospectrum (from now one we only call it 'pseudospectrum') is somewhat trivial: it consists of closed balls of radius ε surrounding the original eigenvalues of DJ. In the case of non-normal matrices however, pseudospectra can be much larger and much more intricate, which is the case we are interested in here.

Local asymptotic stability is determined in the same way for normal and non-normal matrices. The 'spectral abscissa' of DJ is defined as $\alpha(DJ) = \sup_{z\in\Lambda(DJ)}\mathrm{Re}(z)$, where the supremum is the largest real part of $\Lambda(DJ)$; clearly, stability is guaranteed for $\alpha(DJ) < 0$. If DJ is normal, then $||e^{DJt}|| = e^{\alpha(DJ)t}$ and dynamics is determined by $\alpha(DJ)$. The relationship between the spectral abscissa and the system dynamics is evident from the bounds [51]: $e^{\alpha(DJ)t} \leq ||e^{DJt}|| \leq \kappa(\vec{V})e^{\alpha(DJ)t}$ where $\kappa(\mathbf{V}) = ||\mathbf{V}|| \cdot ||\mathbf{V}^{-1}||$ is called the conditioning of the matrix $\mathbf{V}$, which is built from the eigenvectors of DJ, and is a measure of invertibility of $\mathbf{V}$. We see that for a normal dynamics, the conditioning provides an upper bound to the maximum amplification of the perturbations. Clearly, if the spectral abscissa is positive, perturbations are unstable but within the bounds defined above. These bounds can however generalized by introducing the notion of 'ε-pseudospectral abscissa' of DJ. This is defined as $\alpha_\varepsilon(DJ) = \sup_{z\in\Lambda_\varepsilon(DJ)}\mathrm{Re}(z)$, e.g. the largest real part of the spectrum of DJ (for a given ε). The relevance of the ε-pseudospectral abscissa is given by the fact that it provides a lower bound to the maximum amplification of the perturbation [51]:

$$\sup_{\varepsilon\geq 0}\frac{\alpha_\varepsilon(DJ)}{\varepsilon} \leq \sup_{t\geq 0}||e^{DJt}||. \tag{16}$$

The quantity

$$f_{DJ}(\varepsilon) = \frac{\alpha_\varepsilon(DJ)}{\varepsilon} \tag{17}$$

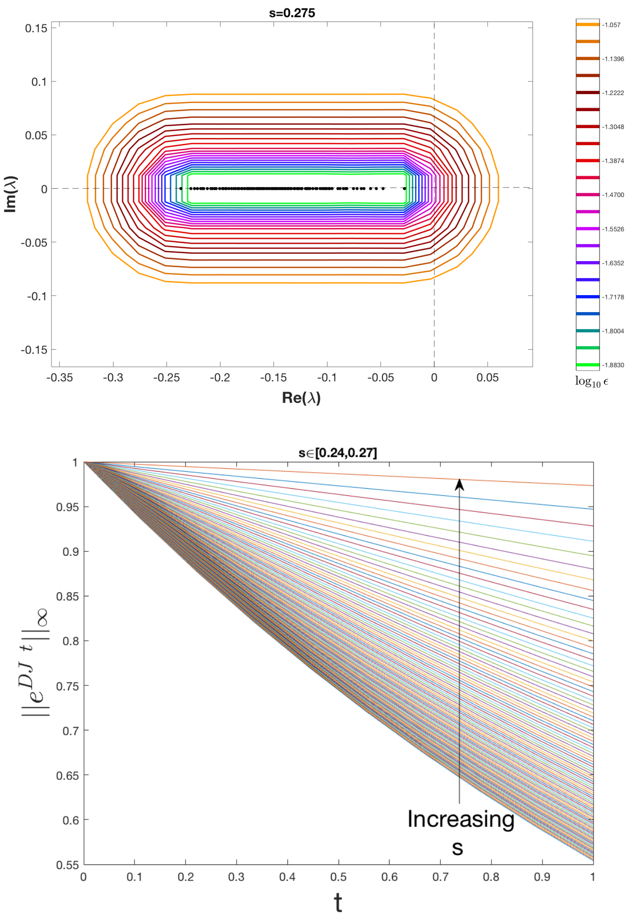

Figure S1: **Jacobian pseudospectra.** Left: ε-Pseudospectrum of the Jacobian matrix for $\varepsilon = [10^{-2}, 10^{-1}]$. Right: behavior of the norm $||e^{DJ\ t}||_\infty$ as a function of time for $s \in [0.24, 0.27]$.

is called the Kreiss constant of the matrix DJ, and is thus relevant in order to understand the transient dynamics for short times. Given the bound of Eqn. (16), it is important to look for values ε^*, at which $f_{DJ}(\varepsilon^*) = 1$. Via a visual analysis of the contours of pseudospectrum, a necessary condition of a critical value of ε^* is that ε^*-contour crosses the imaginary axis. At this point, perturbations from the equilibrium population vector can be in principle amplified, depending on the value of the Kreiss constant.

In order to study the non-normality, we looked at the Jacobian matrix evaluated at the equilibrium points for the dynamics obtained numerically via Monte Carlo. In Fig. S1 (left) we can observe the bulk of ε-pseudospectrum of the Jacobian matrix DJ, fixed Ω as in the main text, and with $\alpha = \beta = 1$ and $\chi = 0.9$. However, such non-normality is not enough to explain the transient instability we observe. A careful numerical analysis of the Kreiss constant for varying s gave us a maximum value of the Kreiss matrix of $K = sup_\varepsilon F_{DK}(\varepsilon) = 0.7$, thus less than 1, the critical value for the amplification. We thus directly studied the norm of e^{DJt} in Fig. S1 (right), not observing any amplification phenomenon up to the instability threshold for the matrix. This implies that $\sup_\varepsilon \frac{\alpha_\varepsilon(DJ)}{\varepsilon} \leq 1$ and that transient instability is not the cause of the escape from the minimum.

D: Basins of attraction

First of all, let us first mention, as shown in the main text, that the tunneling probability depends on the number of dynamical variables. In Fig. 5 of the main text, we see that the larger the height of the barrier (ΔE decreases with s), the larger the number of variables one needs to tunnel through.

In order to obtain a glimpse of the structure of the basins of attractions and the role of the dimensionality in reaching the two asymptotic states we perform the following numerical experiment. Given a random instance of Ω obtained as $\Omega = A^t(AA^t)^{-1}A$, for A a random matrix (with flat probability on $[0,1]$) of size $N_c \times N$ ($N = 200$, $N_c = 100$) we fix χ and $s = \Omega\vec{S}$ in a region in which we know there is a mixing between the local minimum and global minimum of the mean field potential potential $V(x,\chi)$ in $x \in [0,1]$. Specifically, we study the basins of attraction of the system near the mean field situation (e.g. a single memristor), e.g. when many of the variables x_i

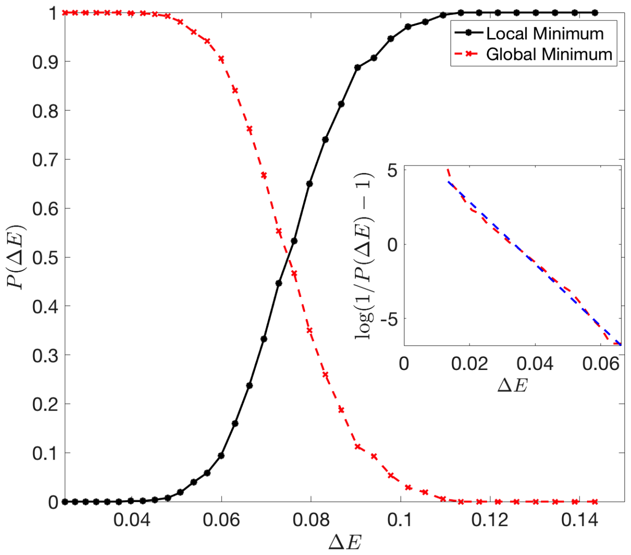

Figure S2: **Effective Fermi distribution of the tunneling.** Distributions $P(x_{cg}^1)$ and $P(x_{cg}^2)$ as a function of ΔE of the potential. Inset: Fit of the Fermi distribution with a chemical potential.

are initialized in the same value. We consider this analysis in the regime reported in the main text, for the values $\chi = 0.9$ and $s \approx 0.22$ which is close to the boundary between the two minima. We then perform the following two experiments. In the first, we choose $x_i(0)$ randomly, and then follow the trajectory and mark it as local or global minimum asymptotic state. Since Ω_{ij} is random and there is no preference between the variables, we choose to plot initial conditions of the variables x_1 and x_2 and mark them depending on which asymptotic state they reached. In the second experiment instead we choose $x_3 \cdots x_N = \tilde{x}$, and x_1 and x_2 still at random, following the procedure of the first experiment and following the trajectory. The value of $\tilde{x}$ is an extra parameter and has to be obtained such that system ends in both minima. A value for $\tilde{x}$ which we found to be working well for random Ω is $\tilde{x} \approx .5135$. The results are shown in Fig. S3, in which we see a complete mixing in the first experiment between the asymptotic states, and a complete separation via a separatrix in the second experiment. The separatrix moves with $\tilde{x}$, and for larger values the system it tends to move towards the global minimum; for smaller values towards the local minimum. Intuitively, this implies that for random initial conditions, for every possible asymptotic state there is a initial condition nearby to the global minimum.

A more careful analysis has been done for random initial conditions. We studied via Monte Carlo sampling the probability distribution, given a random initial condition, of the system being in one or the other local minimum, showing that it can be fit by a Fermi-like distribution depending on the energy barrier ΔE. Let $P(x;s,\chi)$ be the probability distribution as a function of the height of the barrier $\Delta E(s) = E_{max} - E_{local\ min}$ from the local minimum. We obtained such function numerically, averaging over 100 Monte Carlo simulations for each value of s, and shown in Fig. S2. We fit $P(\Delta E)$ with a function of the form $P(x;s,\chi) = 1/(1-e^{-\Delta E(s,\chi)-\mu/T_{eff}})$, where we obtained numerically the value $T_{eff} = 0.0048$, and $\mu = 0.036$. These results are shown in Fig. S2. We see that the transition probabilities of Fig. S2 are consistent with the sigmoidal probabilities described in the main text.

E: Generalization to other window functions and more physically realistic models

Window function

Let us now address here the question whether the phenomenon we described in the main text also occurs within the context of more realistic models. The model we are considering is a polar, current controlled device. For this type of devices there are three effective models used to describe and characterize the nonlinearities in the drift and dopants of the device, and in particular how the model behaves near the boundaries. We consider the Biolek window function $W_p(x,I) = 1-(x-\theta(-I))^{2p}$, the Prodromakis window function, given by $W_p(x) = 1-\left((x-0.5)^2+0.75\right)^p$, the Joglekar window function $W_p(x) = 1-(2x-1)^{2p}$, considered in [40,41]. All these functions act near the boundary, and thus one should expect that exact mean field solution works very well for these models when we do not have a symmetry breaking. In Fig. S4, for instance, we test our prediction on the left and right for the three different models. The exact predictions are not as precise for the dynamics of Biolek's window functions, but are extremely precise for the Joglekar and Prodromakis window functions. The “escape"

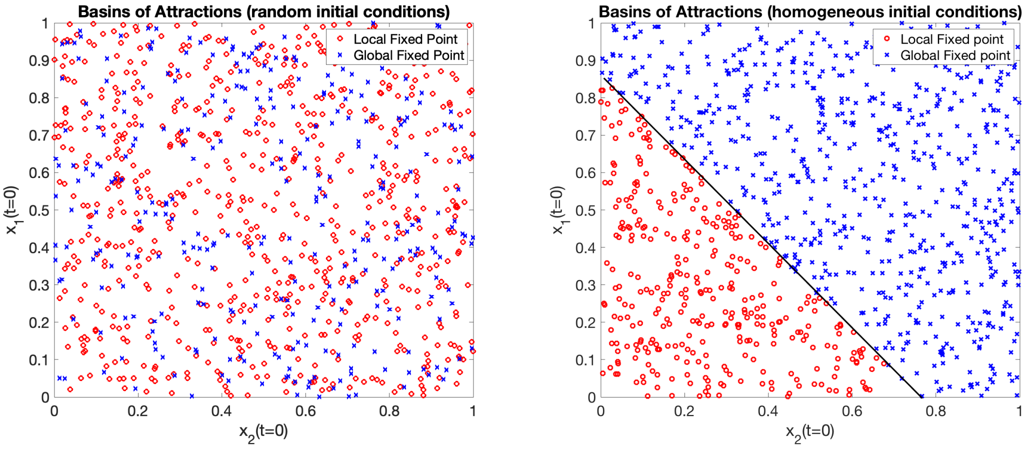

Figure S3: **Basins of attractoin with random and design initial conditions.** Projected basin of attraction for the coarse grained asymptotic states according to the two controlled numerical experiments, as described in the text, in order to visualize the basins of attractions. On the left, we see the completely randomize initial condition, in which we see a complete mixing in the initial conditions for x_1 and x_2 as a function of the asymptotic states. On the right, we see that there is a neat separation between the two asymptotic states via a separatrix bordering two basins of attractions.

from the local minimum occurs in all the three models. Thus, the story we described in the main paper for the simple model applies to these three memristor models as well and is predictive. The key difference is how the memristors approach the boundary. In the main text we have considered sharp boundary window functions, e.g. $W(x) = \theta(x)\theta(1-x)$, but in principle less sharp window function could change the approach to the boundary. This is observed in Fig. S4, but without affecting the key findings of the paper.

Nonlinear current generalization

The mean field analysis can also be applied to other memristor dynamics far more complex than the one we considered in the main paper. Consider the memristor model

$$\frac{dx}{dt} = \frac{1}{\mathscr{T}} \sinh[\frac{I}{i_c}] - \alpha x \tag{18}$$

with $R(x) = R_{off}x + (1-x)R_{on}$, where we have introduced the critical current i_c and the time scale parameter $\mathscr{T}$. The model above is a simplified version of the Pickett's model [38]. For a network of memristors satisfying this dynamical equation, we have

$$\frac{d\vec{x}}{dt} = \frac{1}{\mathscr{T}} \sinh[\frac{\vec{I}}{i_c}] - \alpha\vec{x}. \tag{19}$$

Using the network solution [42] for the currents, we obtain the exact network equation

$$\frac{d\vec{x}}{dt} = \frac{1}{\mathscr{T}} \sinh[\frac{1}{i_c R_{off}}(I-\chi\Omega X)^{-1}\Omega\vec{S}] - \alpha x \tag{20}$$

where Ω and χ are the same as defined in the main text, and where we assume the notation $\sinh(\vec{a})_i = \sinh(a_i)$. Following the same analysis we performed for the linear model, we have a dynamical mean field equation given by

$$\frac{dx_{cg}}{dt} = \frac{1}{\mathscr{T}} \sinh[\frac{1}{i_c R_{off}}(I-\chi x_{cg})^{-1}\bar{s}] - \alpha x_{cg} \tag{21}$$

where again, $x_{cg} = \frac{1}{N}\sum_{ij}\Omega_{ij}x$ and $\bar{s} = \frac{1}{N}\sum_{ij}\Omega_{ij}S_i$, which is an effective mean field dynamics of the system. In the adimensional time variable used before $\tau = \alpha t$, we have again

$$\frac{d}{d\tau}x_{cg} = \frac{1}{\alpha\mathscr{T}} \sinh[\frac{1}{i_c R_{off}}(I-\chi x_{cg})^{-1}\bar{s}] - x_{cg} \equiv -\partial_{x_{cg}}V'(x_{cg}) \tag{22}$$

and where we have introduced the mean field potential

$$V'(x_{cg}) = -\int dx_{cg}\Big(\frac{1}{\alpha\mathscr{T}} \sinh[\frac{1}{i_c R_{off}}(I-\chi x_{cg})^{-1}\bar{s}] - x_{cg}\Big).$$

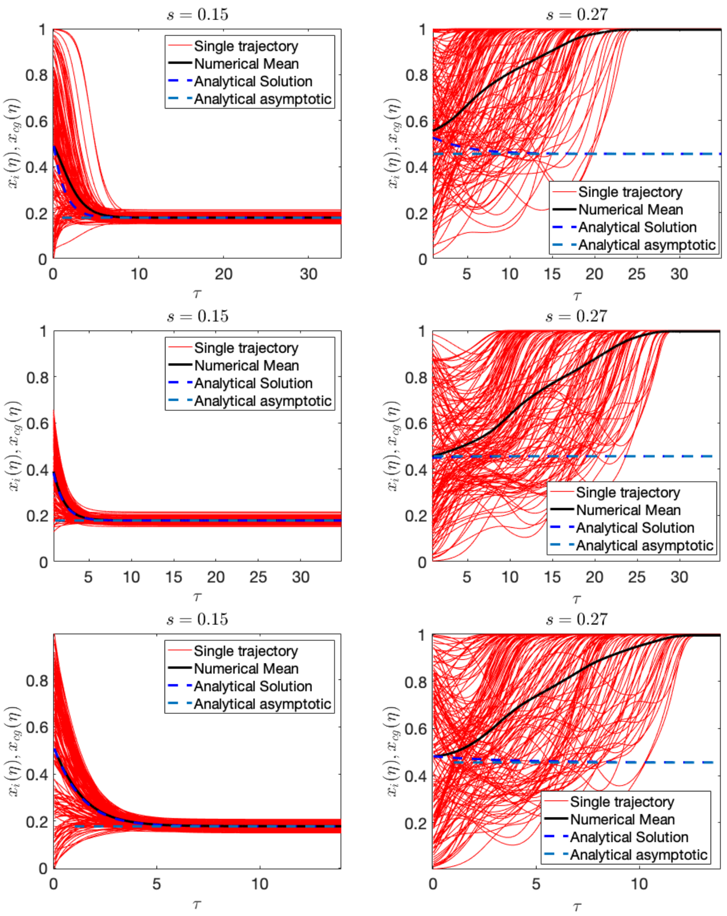

Figure S4: **Results with window functions.** Comparison between the analytical analysis we performed in the paper, and the numerical simulations where we introduced the Biolek window function (top) with $p = 2$, Prodromakis window function with $p = 1$ (center) and Joglekar window functions with $p = 1$.

Such integral can be performed, obtaining

$$V'(x_{cg}) = \frac{\bar{x}^2}{2} + \frac{(1-\chi\bar{x})}{\alpha\mathscr{T}\chi}\sinh\left(\frac{\bar{s}}{i_c R_{\text{off}}\,(1-\chi\bar{x})}\right) - \frac{\bar{s}}{\alpha\mathscr{T}\chi i_c R_{\text{off}}}\text{Chi}\left(\frac{\bar{s}}{(1-\chi\bar{x})\,i_c R_{\text{off}}}\right) \tag{23}$$

where $\text{Chi}(x)$ is the hyperbolic cosine integral, defined as

$$\text{Chi}(x) = \int_0^x \frac{\cosh(t)-1}{t}dt + \log x + \gamma, \tag{24}$$

where γ is the Euler-Mascheroni constant. The potential above is clearly much more complicated than the one of the simple model we considered in the main paper. However, it is not hard to see that the phenomenology of the potential is identical. If we consider $\chi = 0.9, i_c = \alpha = \mathscr{T} = 1, R_{off} = 1$, then the transition occurs for values of the mean voltage $\bar{s}$ within the interval $[0,1]$, but with identical phenomenology: for small values of $\bar{s}$ the potential has a single minimum, while for $s \geq 0.2$ we have a coexistence of two minima and the appearance of a single minimum for $s > 0.27$. While the location of the minimum of the laminar regime is obviously different, the general behavior is qualitatively identical and is shown in Fig. S5.

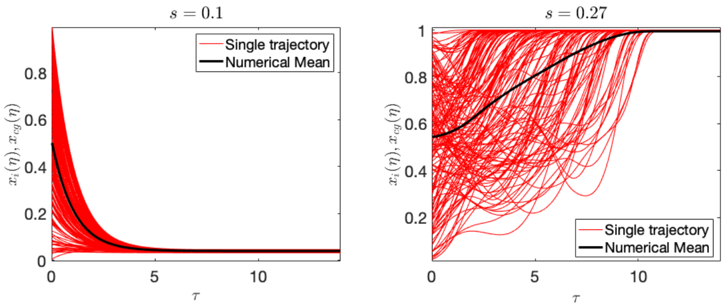

Figure S5: **Results for Sinh memristors.** Transition from the laminar to the rumbling regime for the case of the sinh memristor of eqn. (18).
